# Preconditioning of Human Dental Pulp Stem Cells with Leukocyte- and Platelet-Rich Fibrin-Derived Factors Does Not Enhance Their Neuroregenerative Effect

**DOI:** 10.1155/2019/8589149

**Published:** 2019-04-08

**Authors:** Pascal Gervois, Jessica Ratajczak, Esther Wolfs, Tim Vangansewinkel, Yörg Dillen, Greet Merckx, Annelies Bronckaers, Ivo Lambrichts

**Affiliations:** Morphology Research Group, Biomedical Research Institute, Hasselt University, Diepenbeek, Belgium

## Abstract

Pathologies of the central nervous system are characterized by loss of brain tissue and neuronal function which cannot be adequately restored by endogenous repair processes. This stresses the need for novel treatment options such as cell-based therapies that are able to restore damaged tissue or stimulate repair. This study investigated the neuroregenerative potential of the conditioned medium of human dental pulp stem cells (CM-hDPSCs) on neural stem cell (NSC) proliferation and migration as well as on neurite outgrowth of primary cortical neurons (pCNs). Additionally, the effect of leukocyte- and platelet-rich fibrin (L-PRF) priming on the neuroregenerative potential of the hDPSC secretome on NSCs and pCNs was evaluated. L-PRF contains factors that enhance stem cell-induced regeneration, but its effect on hDPSC-mediated neuroregeneration is unknown. This study demonstrated that CM-hDPSCs enhanced neuritogenesis. Moreover, CM-hDPSCs had a chemoattractant effect on NSCs. Although priming hDPSCs with L-PRF increased brain-derived neurotrophic factor secretion, no additional effects on the paracrine-mediated repair mechanisms were observed. These data support the neuroregenerative potential of hDPSCs, and although priming had no additional effect, the potential of L-PRF-primed hDPSCs on distinct regenerative mechanisms remains to be clarified.

## 1. Introduction

Pathologies of the central nervous system (CNS), such as stroke, are one of the main causes of death and new cases of permanent disabilities which are characterized by tissue damage and loss of brain function [1]. The lost tissue can only be partially reconstituted by the host, for example, via endogenous neural stem cells (NSCs) that migrate towards the injury. However, this regenerative response is ineffective and novel therapies, such as cell-based therapies are needed for treating these disorders. Ideally, NSCs or neural precursor cells (NPCs) would be used as a (stem) cell source as these cells have the potential to differentiate into the majority of neuronal cell types present in the adult brain [2] but due to ethical and practical issues with NSCs, an easily accessible alternative stem cell-source with a neuroregenerative potential is needed.

Human dental pulp stem cells (hDPSCs), neural crest-derived stem cells with mesenchymal stem cell (MSC) characteristics, are an attractive alternative for NSCs or NPCs. Our group and others have previously shown that hDPSCs are characterized by MSC properties as determined by the International Society for Cellular Therapy based on their differentiation potential and surface marker expression [3–6]. In addition to classical multilineage MSC differentiation, we have demonstrated that hDPSCs are capable of neuronal [7] and Schwann cell differentiation [8] *in vitro*. Moreover, it is described that hDPSCs can enhance neuroregeneration via several mechanisms, including cell replacement, neuroprotection, and immunomodulation, and by promoting neuroplasticity and angiogenesis [7, 9–13]. As these mechanisms were mainly thought to be mediated by paracrine actions of the hDPSCs, our group recently investigated the effect of the hDPSC secretome on human SH-SY5Y neuroblastoma cells with encouraging results on hDPSC-driven chemoattraction and neuritogenesis [14]. However, it remains to be determined if these hDPSC-mediated effects can also be observed in cells which are more relevant to the target CNS cells when considering *in vivo* applications of the hDPSC secretome.

Additionally, this study assessed whether preconditioning of hDPSCs enhances their neuroregenerative potential. If hDPSCs are to be considered as a stem cell-based therapy for neurodegenerative disorders, it is important to take the effect of the hypoxic/ischemic and inflammatory microenvironment in which the transplanted cells are situated into account. Several studies have already tried to precondition or “prime” stem cells with hypoxia or with *in vitro* hypoxia mimetics, which was shown to enhance the proangiogenic properties of periodontal ligament stem cells [15] and hDPSCs [16]. Moreover, the trophic effect of hDPSCs on SH-SY5Y cells was enhanced [17], and the secretion of vascular endothelial growth factor (VEGF) was increased [18]. Another approach to prime hDPSCs is to expose them to a cocktail of key components of the inflammatory reaction, which can be found in the blood. Therefore, we evaluated whether leukocyte- and platelet-rich fibrin (L-PRF), a blood-derived biomaterial that is used in the clinic, can enhance the neuroregenerative effect of hDPSCs. L-PRF has shown great promise in wound healing and bone restoration [19, 20], and its therapeutic effect is thought to be mediated by the release of growth factors and cytokines present in this material [21, 22].

In this study, L-PRF is hypothesized to contain multiple (inflammatory) components that modify the stem cell properties and neuroregenerative potential of hDPSCs, an approach that to our knowledge has not been used before. L-PRF is known to contain tumour necrosis factor alpha (TNF-*α*) [23], insulin-like growth factor 1 (IGF-1) [22], and interleukin 1 beta (IL-1*β*) [22] that can influence stem cell characteristics and behaviour. TNF-*α* was shown to enhance the stem cell properties of hDPSCs [24] and the neuronal fate of bone marrow-derived mesenchymal stem cells (BMSCs) [25]. In addition, IGF-1 enhanced the proliferation of BMSCs [26] and TNF-*α* signalling shifted the secretome of adipose-derived stem cells towards a proangiogenic profile [27]. It was demonstrated that IL-1*β* promoted the immunomodulatory properties of human umbilical cord MSCs [28]. Moreover, TNF-*α*-, IGF-1-, and IL-1*β*-stimulated MSCs also increased the expression of CXCR4, a receptor for the chemokine stromal-derived factor-1 (SDF-1). SDF-1 is released by the injured brain which may enhance the migratory capacity of MSCs towards the site of brain injury [25, 26, 28, 29]. MSCs exposed to IL-10 and interferon gamma (IFN-*γ*) producing type 1 regulatory T cells acquire an immunomodulatory phenotype [30], and the secretome of MSCs primed with IL-1*β* or IL-1*α* was shown to reduce the secretion of inflammatory mediators in lipopolysaccharide-activated microglia, demonstrating an immunomodulatory effect of MSC priming [31]. However, the effect of L-PRF on enhancing the neuroregenerative effect of hDPSCs or other MSC subtypes remains elusive.

L-PRF clots are obtained from venous whole blood samples which can be mechanically compressed. During this process, the fluid containing growth factors residing in the clot can be isolated and is termed the L-PRF exudate (EX L-PRF). The factors present in the clot or bound to the fibrin matrix can also be isolated by keeping the clot in culture after which the conditioned culture medium (CM L-PRF) can be collected which then contains the factors that are being released over time.

This study was aimed at evaluating paracrine-mediated effects of hDPSCs on mouse NSCs and primary cortical neurons (pCNs). The influence of the hDPSC secretome on NSC proliferation and migration was assessed. Moreover, the influence of the hDPSC factors on neurite outgrowth of pCNs was investigated. In order to evaluate the effect of L-PRF priming on hDPSCs, the optimal priming conditions were determined. hDPSCs were exposed to various concentrations of EX/CM L-PRF to investigate the mitogenic and metabolic effect of L-PRF on hDPSCs. Moreover, a growth factor array was used to determine the composition of both L-PRF subfractions. Additionally, hDPSCs were exposed to EX/CM L-PRF in different concentrations for various time points in order to evaluate enhanced growth factor production by hDPSCs. Brain-derived neurotrophic factor (BDNF) secretion was used as a measure for enhanced neurotrophin production as this factor was previously shown to be secreted by hDPSCs [7, 32] and was demonstrated to enhance functional recovery and neurogenesis [33, 34]. Afterwards, the conditioned medium of primed hDPSCs (prCM-hDPSCs) was used in the assays on NSCs and pCNs (*vide supra*) in parallel with CM-hDPSCs to evaluate the effect of L-PRF priming on hDPSC-mediated mechanisms of neuroregeneration.

## 2. Materials and Methods

### 2.1. Isolation and Cell Culture of hDPSCs

Human dental pulp tissue was obtained from patients of both genders aged 14 to 26 (*n* = 11) undergoing routine extraction of third molars for orthodontic reasons with informed consent of the patient or after approval of the legal guardian. This study was approved by the Medical Ethical Committee of Hasselt University (13/0104u). All experiments were performed in accordance with the guidelines and regulations of the host institution. Subsequently, hDPSCs were isolated by the explant method as described previously [4, 5, 7]. Briefly, the tooth was fractioned mechanically after which the dental pulp tissue was isolated with sterile forceps. Afterwards, the tissue was rinsed in standard hDPSC medium which consists of alpha modification of minimum essential medium (*α*-MEM) supplemented with 2 mM L-glutamine, 100 U/ml penicillin, 100 *μ*g/ml streptomycin, and 10% foetal bovine serum (FBS). Next, the pulp was cut into small fragments of 1-2 mm^3^ which were grown in 6-well plates and incubated at 37°C in a humidified atmosphere containing 5% CO_2_. The culture medium was changed every 3-4 days and after reaching 70-80% confluency, the cells were harvested using 0.05% trypsin with ethylenediaminetetraacetic acid (EDTA). For further expansion, hDPSCs were seeded at a density of 4 × 10^3^ cells/cm^2^ and the cell culture was evaluated on a regular basis with a Nikon Eclipse TS100 inverted phase contrast microscope equipped with a Jenoptik ProgRes C3 camera (Jenoptik, Jena, Germany) with corresponding ProgRes Capture Pro 2.7 software. All experiments were conducted with hDPSCs between passages two and eight. All chemicals, supplements, and media were purchased from Sigma-Aldrich (St. Louis, MO, USA) unless stated otherwise.

### 2.2. Isolation and Culture of L-PRF

**Figure 1:**
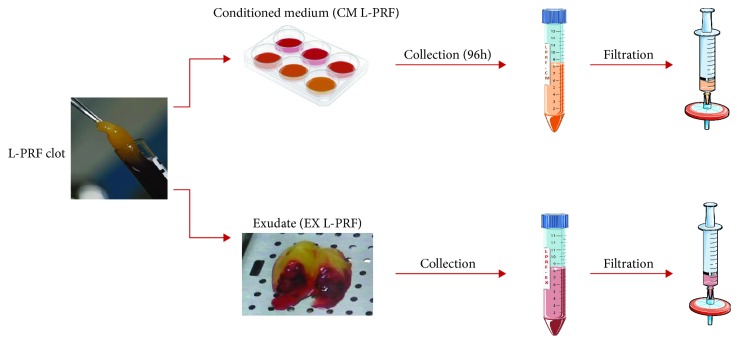
Workflow to isolate L-PRF subfractions. The L-PRF clot is isolated by centrifugation from blood samples that are subsequently processed for CM L-PRF and EX L-PRF isolation. L-PRF clots are transferred to 6-well plates in culture medium, and CM L-PRF was collected after 96 h. The L-PRF clot is transferred to the Xpression™ Box and by gravitational compression of the clot, EX L-PRF is collected. Both CM- and EX L-PRF were filtered before use. This figure was partly created using Servier Medical Art licensed under a Creative Common Attribution 3.0 Generic License, available online at http://smart.servier.com/.

L-PRF was isolated from healthy donors of both genders, aged 25 to 54 (*n* = 10). Isolation was approved by the medical ethical committee of Hasselt University and the Clinical Trial Center from KU Leuven (S58789/B322201628215/I/U). L-PRF was prepared according to the guidelines of the IntraSpin™ Centrifuge (Intra-Lock International, Boca Raton, FL, USA). Briefly, 9 ml venous blood samples were collected in glass-coated plastic tubes (VACUETTE® Z Serum Clot Activator Tubes; Greiner Bio-One, Kremsmünster, Austria) and spun down for 12 min at 400 g. Afterwards, L-PRF clots were collected and coagulated red blood cells were removed. The resulting clots were then transferred to the Xpression™ Box (Intra-Lock International), and a weighted press was put onto the clots. This device extracts exudate (EX) L-PRF from the clot in a controlled manner and results in a thin compressed layer of L-PRF with consistent thickness. The exudate was sterile filtered, aliquoted, and stored at -80°C for later use. L-PRF clots were transferred to 6-well plates and put in culture in alpha modification of minimum essential medium (*α*-MEM) without FBS but containing 2 mM L-glutamine, 100 U/ml penicillin, and 100 *μ*g/ml streptomycin for 96 h. Afterwards, the CM L-PRF was collected, sterile filtered, aliquoted, and stored at -80°C.  Figure 1 provides a schematic overview that describes CM L-PRF and EX L-PRF preparation.

### 2.3. Human Cytokine Antibody Array

A cytokine antibody array (ab133998; Abcam, Cambridge, UK) was performed on CM and EX L-PRF (*n* = 4) at a protein concentration of 10 mg/ml according to the manufacturer's instructions. Protein concentrations of the samples were determined with a bicinchoninic acid assay (BCA; Thermo Fisher Scientific, Waltham, MA, USA) following the user manual. Relative pixel density was measured using Fiji software to compare relative protein levels between EX and CM L-PRF. This analysis was previously performed by our research group [35]. This study reinterpreted the data of these arrays focusing on the inflammatory factors present in both L-PRF subfractions.

### 2.4. Metabolic Activity and Proliferation of hDPSCs Exposed to L-PRF

The relative metabolic and mitotic activities of hDPSCs exposed to CM/EX L-PRF (*n* = 5) were evaluated by means of a 3-(4,5-dimethylthiazol-2-yl)-2,5-diphenyltetrazolium bromide (MTT) and propidium iodide (PI) assay, respectively. The latter takes the amount of PI intercalating between DNA as a measure for the total number of cells. The average values of 3 different hDPSC donors were calculated, primed with 5 different L-PRF donors in 5 independent experiments. hDPSCs were seeded in triplicate at a density of 3 × 10^4^ cells/cm^2^ in 96-well plates in standard hDPSC medium. After 24 h, the medium was discarded and hDPSCs were exposed to 1%, 2%, 5%, and 10% CM/EX L-PRF for 24 h, 48 h, or 72 h. hDPSCs exposed to hDPSC standard medium without FBS and with 10% FBS were used as a negative and positive control, respectively. To perform the MTT assay at each time point, the medium was removed and 500 *μ*g/ml MTT was added. After 4 h of incubation, the MTT-containing solution was removed and 0.01 M of glycine in DMSO was added to the wells in order to dissolve the formed formazan crystals. The absorbance was measured at 570 nm and corrected for the background signal at 655 nm with an iMark™ Microplate Reader (Bio-Rad).

The PI assay was performed by removing the medium and adding 75 *μ*l of lysis buffer to each well (ChemoMetec, Allerod, Denmark) after which 75 *μ*l of stabilizing solution (ChemoMetec) containing PI (10 *μ*g/ml) was added and incubated at room temperature in the dark for 15 min. Afterwards, the solution was transferred to a black 96-well plate and fluorescence was excited at 540 nm and measured at 612 nm with a Fluostar Optima (BMG Labtech, Ortenberg, Germany). All data were normalized to the values of hDPSCs exposed to standard hDPSC medium without FBS after 24 h exposure.

### 2.5. Priming of hDPSCs with L-PRF Exudate and Conditioned Medium

hDPSCs from two different donors were primed with four different CM/EX L-PRF donors in various concentrations (1%, 5%, and 10%) in standard hDPSC medium without FBS. Cells were seeded at 1.5 × 10^4^ cells/cm^2^ in standard hDPSC medium. After plastic adherence, the medium was removed and serum-free hDPSC medium supplemented with the appropriate concentration of CM/EX L-PRF was added to prime the cells. After 24 h or 48 h, this priming medium was removed and the cells were thoroughly washed with phosphate-buffered saline (PBS). Next, serum-free Neurobasal-A medium (Invitrogen; Carlsbad, CA, USA) supplemented with 2 mM L-glutamine, 100 U/ml penicillin, and 100 *μ*g/ml streptomycin was added to the hDPSCs. 48 h later, the medium was collected, centrifuged at 300 g for 6 min, aliquoted, and stored at -80°C for later use. The cell number of all conditions was determined with a Moxi cell counter (ORFLO Technologies, Ketchum, ID, USA).

The inductive effect of EX L-PRF and CM L-PRF on the growth factor production of hDPSCs was determined by means of a BDNF ELISA according to the instructions of the manufacturer (RayBiotech®; Norcross, GA, USA). The BDNF concentration was normalized to 1 × 10^5^ cells to correct for variations in cell number due to the priming conditions.

### 2.6. Preparation of Conditioned Medium of hDPSCs and hDPSCs Primed with L-PRF

In order to prepare the conditioned medium of primed (prCM-hDPSCs) and nonprimed hDPSCs (CM-hDPSCs), cells (*n* = 7 hDPSC donors) were seeded at a density of 1.4 × 10^4^ cells/cm^2^ in standard culture medium. After the cells adhered to the surface, the medium was changed to 1 ml/5 cm^2^ of serum-free hDPSC medium with or without 10% EX L-PRF from 3 different donors. 48 h later, the cells were rinsed with PBS and the medium was changed to serum-free Neurobasal-A medium, supplemented with 2 mM L-glutamine, 100 U/ml penicillin, and 100 *μ*g/ml streptomycin for 48 h to allow EX L-PRF-induced secretion of growth factors. After 48 h, the medium was collected, centrifuged at 300 g, aliquoted, and stored at -80°C for later use. In addition, the cells were counted to later normalize prCM-hDPSC volumes to corresponding CM-hDPSC donors for the increased cell numbers of exudate-exposed cells compared to controls. All prCM-hDPSC samples were prepared in parallel with CM-hDPSCs. For each experiment, the average experimental output value of the effect of prCM-hDPSCs was calculated and compared to CM-hDPSC. prCM-hDPSCs was collected from hDPSCs primed with 3 different L-PRF donors which were both kept consistent within each experiment.

### 2.7. Isolation and Culture of Mouse NSCs

Mouse NSCs were isolated from 6 foetal brains as described previously by Conti et al. [36] with minor modifications based on Reekmans et al. [37]. At gestational days 14-15, pregnant C57BL/6JOlaHsd mice (Envigo, Cambridgeshire, UK) were sacrificed by cervical dislocation and the foetuses were removed from the abdomen as approved by the ethical commission of Hasselt University (201410K). All experiments were conducted in accordance with the guidelines and regulations of the institute. Subsequently, the brains were removed and cut into small fragments in cold (4°C) PBS with 100 U/ml penicillin and 100 *μ*g/ml streptomycin. The brain fragments were then transferred to a new vial and centrifuged for 8 min at 200 g. Afterwards, the supernatant was removed and incubated with 0.2% collagenase A (Roche, Basel, Switzerland) and DNase-I (2000 Kunitz units/50 ml) in PBS for 1.5 h at 37°C. The obtained dissociated tissue was subsequently washed and resuspended in Neurobasal-A medium supplemented with 1% N2 (Invitrogen), 10 ng/ml epidermal growth factor (EGF) and basic fibroblast growth factor (bFGF) (both from ImmunoTools, Friesoythe, Germany), 100 U/ml penicillin, and 100 *μ*g/ml streptomycin. The cell suspension was then rinsed through a 70 *μ*m cell strainer and transferred to an uncoated culture flask to allow neurosphere formation and removal of unwanted, plastic adherent cells. The neurospheres were allowed to grow for 4-5 days at 37°C in a humidified atmosphere containing 5% CO_2_, and the growth factors were replenished every other day. Neurosphere collection occurred by centrifugation at 200 g for 6 min followed by dissociation with Accutase for 5 min. Subsequently, the obtained NSCs were seeded at 2.5 × 10^4^ cells/cm^2^ on a 5 *μ*g/ml fibronectin- (R&D Systems, Minneapolis, MN, USA) coated surface in Neurobasal-A medium supplemented with 2% B27 without vitamin A (Invitrogen), 10 ng/ml EGF and bFGF, 2 mM L-glutamine, 100 U/ml penicillin, and 100 *μ*g/ml streptomycin which will be referred to as standard NSC medium. The culture medium was changed every 3 to 4 days and cells were subcultured when 70-80% confluence was reached. NSCs were harvested by incubation with Accutase for 5 min (37°C) and centrifuged for 5 min at 300 g. For immunocytochemical (ICC) analysis, the NSCs were seeded at 2 × 10^4^ cells/cm^2^ on fibronectin-coated glass coverslips or on coated Thermanox® plastic coverslips for transmission electron microscopy (TEM) processing. Immunophenotyping of all NSC lines (*n* = 6) was performed for the markers described in Reekmans et al. [37]. One representative NSC line was used in the subsequent experiments.

### 2.8. Transwell Migration Assay

Tissue culture inserts (ThinCert™, 8 *μ*m pore size, Greiner Bio-One) were coated with 5 *μ*g/ml fibronectin and seeded with NSCs (5 × 10^4^ cells/insert) in Neurobasal-A medium containing 0.2% B27, 2 mM L-glutamine, 10 ng/ml bFGF and EGF, 100 U/ml penicillin, and 100 *μ*g/ml streptomycin. The bottom chemoattractant compartment of the well plate contained either CM-hDPSCs or prCM-hDPSCs (*n* = 4) to which 0.2% B27 and 10 ng/ml EGF and bFGF were freshly added. As a negative (nonchemoattractant) control, Neurobasal-A medium containing 0.2% B27, 2 mM L-glutamine, 10 ng/ml bFGF and EGF, 100 U/ml penicillin, and 100 *μ*g/ml streptomycin was added. The same medium containing 2% B27, 10 ng/ml EGF and bFGF, and 100 ng/ml SDF-1 (ImmunoTools) was used as a positive control. After 24 h, the transmigrated cells were fixed with 4% paraformaldehyde (PFA) and stained with 0.1% crystal violet. Data were collected from 4 independent experiments, and two representative micrographs were taken per insert. Migration was quantified with AxioVision software (Carl Zeiss, Aalen, Germany).

### 2.9. Metabolic Activity and Proliferation of NSCs

The metabolic and mitotic activities of NSCs exposed to CM-hDPSCs or prCM-hDPSCs (*n* = 6) were evaluated by means of 6 independent MTT and PI assays, respectively. NSCs were seeded in 96-well plates at a density of 1.5 × 10^4^ cells/cm^2^ in standard NSC medium. After 24 h, the cells were exposed to the experimental conditions and both assays were performed 24 h, 48 h, and 72 h later as described previously (*vide supra*). To all experimental conditions, 0.2% B27 and 10 ng/ml EGF and bFGF were added. As internal controls, NSCs were exposed to standard NSC medium with 0.2% B27 instead of 2% and standard NSC medium with 20 ng/ml of EGF and bFGF to serve as a negative and positive control, respectively. All data were normalized to NSCs exposed to standard NSC medium with 0.2% B27 after 24 h exposure.

### 2.10. Isolation of Primary Cortical Neurons

Mouse pCNs were isolated from foetal brains (*n* = 46) by an adapted protocol, based on [38]. At gestational days 17-18, pregnant C57BL/6JOlaHsd mice (Envigo) were sacrificed by cervical dislocation and the foetuses were removed from the abdomen as approved by the ethical commission of Hasselt University (201410K). All experiments were conducted in accordance with the guidelines and regulations of the institute. Subsequently, the brains were removed and put into preheated Hank's balanced salt solution (HBSS; Invitrogen) supplemented with 7 mM 4-(2-hydroxyethyl)-1-piperazineethanesulfonic acid (HEPES; Invitrogen), 100 U/ml penicillin, and 100 *μ*g/ml streptomycin. Afterwards, the meninges were carefully removed with forceps under a Leica S6E stereomicroscope (Leica Microsystems, Wetzlar, Germany) and the cortex was dissected clear from the hippocampus, the thalamus, and the striatum. The obtained cortices were collected in HBSS/HEPES and incubated with 0.05% trypsin for 15 min at 37°C. The cortices were then washed three times with minimum essential medium (MEM; Invitrogen) supplemented with 10% horse serum (Invitrogen), 0.6% glucose, 100 U/ml penicillin, and 100 *μ*g/ml streptomycin and were mechanically dissociated. The acquired cell suspension was centrifuged for 8 min at 300 g, and the cells were resuspended in supplemented MEM medium and rinsed through a 70 *μ*M cell strainer to obtain a single-cell suspension. Neurons were seeded at 2.5 × 10^4^ cells/cm^2^ on coverslips previously coated with 20 *μ*g/ml poly-d-lysine (Corning; Corning, NY, USA).

### 2.11. Immunocytochemistry

Cells seeded on glass coverslips were fixed in 4% PFA, and immunostainings were performed according to a standardized protocol [4, 7]. In summary, in the case of an intracellular epitope, cells were permeabilized with 0.05% Triton X-100 in PBS at 4°C for 30 minutes. Afterwards, 10% donkey serum was used to block nonspecific binding sites. Cells were incubated at room temperature for 1 hour with the primary antibodies listed in Table 1. Afterwards, cells were incubated with the appropriate secondary antibody at room temperature for 30 minutes. Nuclei were counterstained with 4,6-diamidino-2-phenylindole (DAPI), and coverslips were mounted with antifade mounting medium (Dako, Glostrup, Denmark) on glass slides. Negative controls were included in each staining in which the staining procedure was performed in parallel with the other samples but with omission of the primary antibody. Micrographs were taken with a Nikon Eclipse 80i fluorescence microscope equipped with a 2MBWc digital sight camera and NIS-Elements software.

### 2.12. Transmission Electron Microscopy

Cells cultured on plastic Thermanox® coverslips were prepared for ultrastructural analysis as previously described [4]. Briefly, postfixation of cells fixed with 2% glutaraldehyde was performed with 2% osmium tetroxide in 0.05 M sodium cacodylate buffer (pH = 7.3) at 4°C for 1 hour. Dehydration of the samples was performed by ascending concentrations of acetone. The dehydrated samples were impregnated overnight in a 1 : 1 mixture of acetone and araldite epoxy resin at room temperature. After impregnation, samples were embedded in araldite epoxy resin at 60°C using the pop-off method. Embedded samples were cut in slices of 40-60 nm with a Leica EM UC6 microtome (Leica, Wetzlar, Germany) and transferred to 0.7% formvar-coated copper grids (Aurion, Wageningen, the Netherlands). The samples were contrasted with 0.5% uranyl acetate and a stabilized solution of lead citrate using a Leica EM AC20 (Leica). TEM analysis was performed with a Philips EM208 S electron microscope (Philips, Eindhoven, the Netherlands) equipped with a Morada Soft Imaging System camera with corresponding iTEM-FEI software (Olympus SIS, Münster, Germany).

### 2.13. Neuritogenesis Assay of pCNs

After freshly isolated pCNs (2.5 × 10^4^ cells/cm^2^) adhered to the glass coverslips, the plating medium (minimum essential medium supplemented with 10% horse serum (Invitrogen), 0.6% glucose, 100 U/ml penicillin, and 100 *μ*g/ml streptomycin) was changed to CM-hDPSCs or prCM-hDPSCs (*n* = 5) to which 2% B27 with Vitamin A was freshly added. pCNs in standard pCN medium were used as controls. After 72 h, the pCNs were fixed with 4% PFA and an ICC staining was performed for *β*-III tubulin. Automated Neurite Outgrowth Assay 6.1 software (NEO 6.1; DCI Labs, Keerbergen, Belgium) was applied to evaluate neurite length and the number of neurites per pCN. The mean length of the longest neurite was calculated with Fiji software [39] with the NeuronJ plugin [40]. For each sample analysed with Fiji, at least 50 neurites were counted and only clearly distinguishable neurites were measured. For prCM-hDPSCs, the average neurite length of primed CM of 5 different hDPSC donors was calculated, primed with 3 different L-PRF donors. Simultaneously, the fraction of *β*-III tubulin-positive cells was evaluated to determine the neuronal purity of the obtained pCN culture. As an indication for network formation between pCNs exposed to CM-hDPSCs and prCM-hDPSCs, the number of cells making contact with one or more neighbouring cells in *β*-III tubulin-stained pCNs was evaluated with Fiji software. All experiments were performed on cultures of 3 independent pCN isolations.

### 2.14. Statistical Analysis

Statistical analysis was performed using GraphPad Prism 7 software (GraphPad, San Diego, CA, USA). Normality was checked with the Shapiro-Wilk test. Normal distributed data were tested with one-way ANOVA and Bonferroni's multiple comparison posttest. Nonparametric data were analysed with the Kruskal-Wallis test followed by Dunn's test. Differences were considered statistically significant at *p* values ≤ 0.05. Data were expressed as mean ± standard error of the mean (SEM).

## 3. Results

### 3.1. Characterization of the L-PRF Secretome

**Figure 2:**
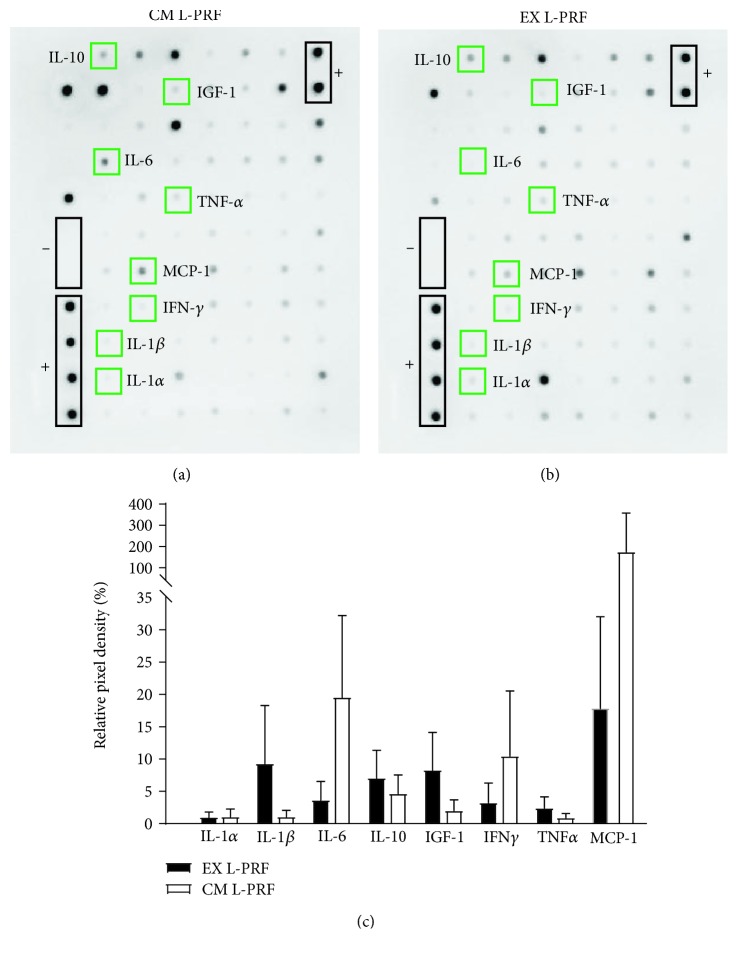
Protein release profile of EX and CM L-PRF. Representative antibody array of the proteins released from CM L-PRF (a) and EX L-PRF (b) (*n* = 4). Semiquantitative analysis using relative pixel density (c) was performed to compare relative protein levels between CM and EX L-PRF. Both CM L-PRF (a) and EX L-PRF (b) contained several inflammatory mediators, including IL-1*α*, IL-1*β*, IL-6, IL-10, IGF-1, IFN-*γ*, TNF-*α*, and MCP-1. None of these mediators were significantly more abundant in either L-PRF fraction. Data are presented as mean ± SEM.

The growth factors in the different L-PRF fractions (*n* = 4) were investigated using an antibody array (Figure 2), which was previously used by our research group and published in Ratajczak et al. [35]. A reinterpretation of these arrays focusing on the inflammatory factors is presented in this study. Both CM L-PRF (Figure 2(a)) and EX L-PRF (Figure 2(b)) contained several inflammatory mediators which were shown to influence stem cell characteristics, including IL-1*α*, IL-1*β*, IL-6, IL-10, IGF-1, IFN-*γ*, TNF-*α*, and monocyte chemotactic protein-1 (MCP-1), albeit with high donor variability. Therefore, multiple L-PRF donors were included in subsequent priming experiments. Semiquantitative analysis of the relative pixel density of these proteins of interest suggested that none of these mediators were more abundant in either L-PRF fractions (Figure 2(c)) as depicted by green squares in Figures 2(a) and 2(b).

### 3.2. CM and EX L-PRF Stimulated hDPSC Proliferation

**Figure 3:**
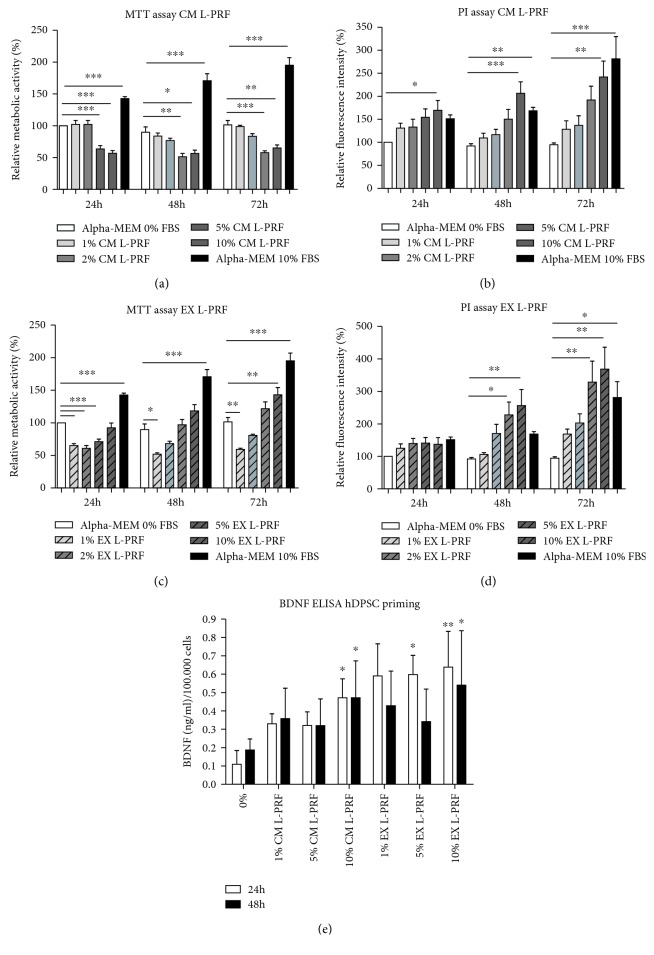
Metabolic and proliferative activity of hDPSCs exposed to CM and EX L-PRF and the effect of L-PRF priming on hDPSC-derived BDNF secretion. The metabolic (a, c) and proliferative (b, d) effect of hDPSCs that were exposed for 24 h, 48 h, and 72 h to CM (a, b) or EX L-PRF (c, d) was evaluated by means of an MTT and PI assay, respectively (*n* = 5). CM and EX L-PRF had an inverse effect on hDPSC metabolism as high concentrations of CM significantly diminished, and high concentrations of EX L-PRF significantly stimulated hDPSC metabolism. Both CM and EX L-PRF had a dose-response effect on proliferation as higher concentrations significantly enhanced hDPSC proliferation. BDNF secretion (e) was increased in hDPSCs primed (*n* = 4) with 10% CM L-PRF or 5% and 10% EX L-PRF for 24 h while significant BDNF secretion could only be observed when hDPSCs were primed for 48 h with 10% CM or EX L-PRF. ^∗^*p* value ≤ 0.05, ^∗∗^*p* value ≤ 0.01, and ^∗∗∗^*p* value ≤ 0.001. Data are expressed as mean ± SEM.

In order to evaluate the influence of CM and EX L-PRF on hDPSC metabolism and proliferation, an MTT and PI test was performed 24 h, 48 h, and 72 h after exposure (Figure 3). CM L-PRF had an inverse effect on hDPSC metabolism (Figure 3(a)) and hDPSC proliferation (Figure 3(b)). Higher concentrations of CM L-PRF (5% and 10%) significantly decreased hDPSC metabolism at every time point down to 51.25%-65.05% of the reference values measured after 24 h (^∗^*p* values ≤ 0.05, ^∗∗^*p* ≤ 0.01, and ^∗∗∗^*p* ≤ 0.001). On the other hand, 10% CM L-PRF significantly stimulated (*p*- value ≤ 0.01) hDPSC proliferation up to 2.5-fold after 72 h. Low concentrations of EX L-PRF (1%, 2%, and 5%) significantly decreased hDPSC metabolism down to 60.87%±9.538 after 24 h (*p* value ≤ 0.001), but this effect was diminished 48 h and 72 h later where only 1% EX L-PRF significantly decreased hDPSC metabolism (^∗^*p* value ≤ 0.05; ^∗∗^*p* ≤ 0.01) (Figure 3(c)). The highest concentration of EX L-PRF (10%) significantly increased (^∗∗^*p* value ≤ 0.01) metabolism 1.43-fold after 72 h. When cell numbers were evaluated with PI, no decrease in cell numbers could be observed. While no effect was observed after 24 h, 5% and 10% EX L-PRF significantly enhanced hDPSC proliferation (^∗^*p* value ≤ 0.05; ^∗∗^*p* ≤ 0.01) after 48 h and 72 h (Figure 3(d)). 10% EX L-PRF increased hDPSC proliferation 3.7-fold, while the positive control increased hDPSC number 2.7-fold.

### 3.3. Priming of hDPSCs with L-PRF Altered Their BDNF Secretion

The CM of hDPSCs primed with various concentrations of CM and EX L-PRF for 24 h or 48 h was collected and subjected to an ELISA for BDNF as a measure for neurotrophin production (Figure 3(e)). 24 h priming with 10% CM L-PRF (*p* value ≤0.01) and 5% and 10% EX L-PRF (^∗^*p* value ≤ 0.05; ^∗∗^*p* ≤ 0.01) significantly enhanced BDNF production from 0.1 ng/ml ± 0.15 to maximum 0.64 ng/ml ± 0.39 per 1 × 10^5^ hDPSCs compared to nonprimed (hDPSCs exposed to *α*-MEM with 0% FBS). A significant increase in BDNF production after 48 h priming could only be observed when hDPSCs were primed with 10% CM or EX L-PRF (^∗^*p* value ≤ 0.05), increasing the BDNF concentration from 0.19 ng/ml ± 0.12 to 0.54 ng/ml ± 0.59 per 1 × 10^5^ hDPSCs.

### 3.4. Morphological and Immunocytological Characteristics of NSCs

**Figure 4:**
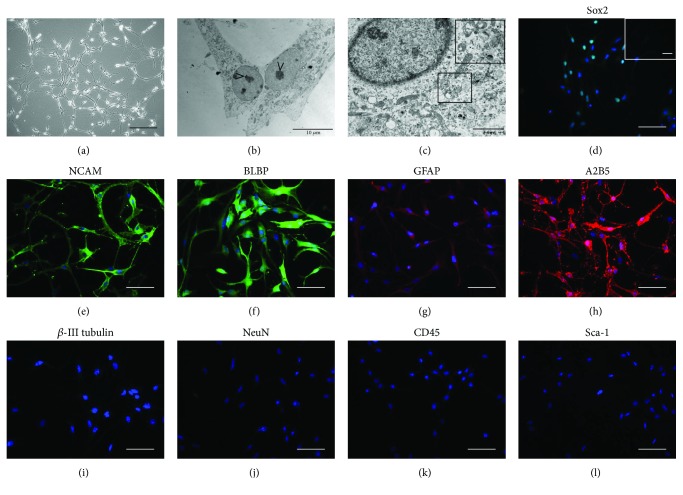
NSC characterization. NSC cultures were characterized by a large perikaryon and intercellular extensions (a). Ultrastructurally, NSCs were characterized by a large nucleolus (b) and by a cytoplasm rich in MVBs (c, insert). NSCs showed immunoreactivity for the NSC markers Sox2 (d), NCAM (e), BLBP (f), GFAP (g), and A2B5 (h). No reactivity was observed for *β*-III tubulin (i), NeuN (j), CD45 (k), and Sca-1 (l). Images show representative micrographs of NSCs isolated from 6 different foetuses. Scale bars: a: 200 *μ*m; b: 10 *μ*m; c: 2 *μ*m. Scale bars: d–l: 50 *μ*m.

Neurosphere-cultured NSCs were dissociated and the single-cell suspension was transferred to fibronectin-coated flasks to allow adherent cell culture. Cells presented with a large perikaryon with peripheral halo that typically acquired a bipolar or multipolar morphology with intercellular extensions (Figure 4(a)). Ultrastructurally, these cells were characterized by a large nucleus with prominent nucleolus (Figure 4(b)) and the cytoplasm contained several multivesicular bodies (MVBs) (Figure 4(c), insert).

NSCs were immunophenotyped for the NSC markers Sox2, NCAM, BLBP, GFAP, and A2B5 (Figures 4(d)–4(h)). The obtained NSCs did not express markers of a more mature neuronal phenotype such as *β*-III tubulin and NeuN (Figures 4(i) and 4(j)) and did not express the hematopoietic marker CD45 and the mesenchymal marker Sca-1 (Figures 4(k) and 4(l)).

### 3.5. The hDPSC Secretome Acts as a Chemoattractant on NSCs

**Figure 5:**
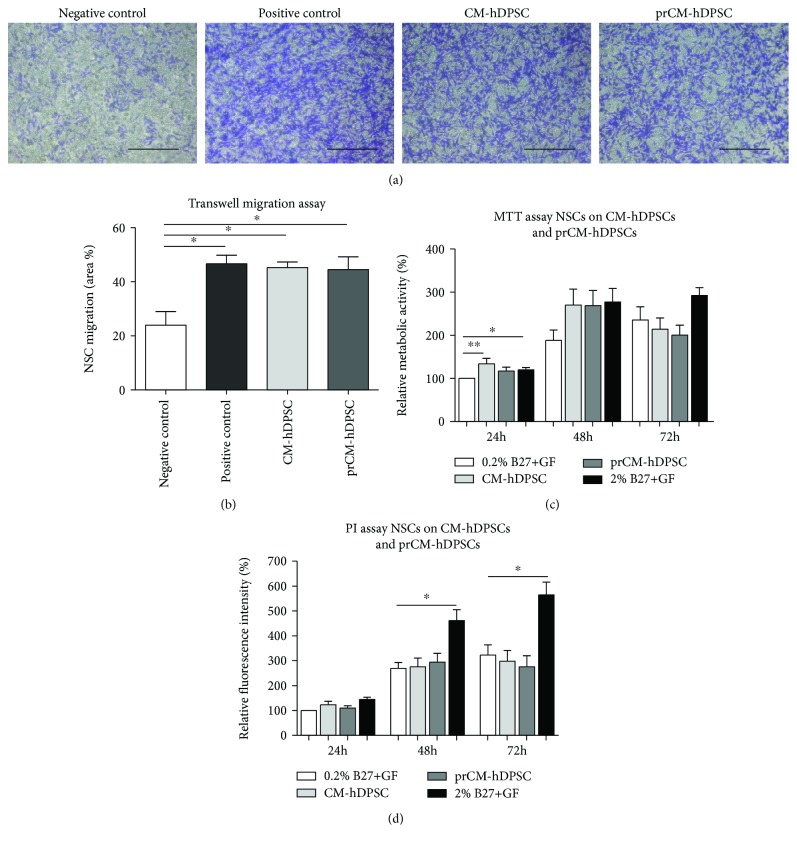
The effect of CM-hDPSCs and prCM-hDPSCs on NSC chemoattraction, metabolic activity, and proliferation. Transwell inserts stained with 0.1% crystal violet to demonstrate CM-hDPSCs and prCM-hDPSCs induced NSC chemoattraction (a) and showed that both CM-hDPSCs and prCM-hDPSCs (*n* = 4) attracted NSCs, but priming did not significantly increase this effect (b). The effect of CM-hDPSCs and prCM-hDPSCs (*n* = 6) on NSC metabolism and proliferation was evaluated by means of an MTT and PI assay, respectively. CM-hDPSCs and prCM-hDPSCs did not significantly influence NSC metabolism (c) or proliferation (d) after 24 h, 48 h, and 72 h although CM-hDPSCs significantly stimulate NSC metabolism after 24 h. No significant difference could be observed between CM-hDPSCs and prCM-hDPSCs. ^∗^*p* value ≤ 0.05 and ^∗∗^*p* value ≤ 0.01. Data are expressed as mean ± SEM. Scale bars: a: 200 *μ*m.

The chemoattractant properties of primed- and nonprimed hDPSCs were evaluated by means of a transwell migration assay. Representative micrographs of 0.1% crystal violet inserts of the experimental conditions are shown in Figure 5(a). Both CM-hDPSCs and prCM-hDPSCs significantly attracted NSCs after 24 h incubation (^∗^*p* value ≤ 0.05) (Figure 5(b)). No significant difference was detected between CM-hDPSCs and prCM-hDPSCs.

### 3.6. CM-hDPSCs and prCM-hDPSCs Had No Effect on Mitotic and Metabolic Activity of NSCs

The effect of CM-hDPSCs and prCM-hDPSCs on NSC metabolism and proliferation was evaluated by means of an MTT and PI assay, respectively. CM-hDPSCs significantly stimulated NSC metabolism after 24 h exposure (^∗∗^*p* value ≤ 0.01), and although there appears to be a metabolism-stimulating effect after 48 h, this was not significant (Figure 5(c)), and the trend disappeared after 72 h exposure. Similarly, CM-hDPSCs and prCM-hDPSCs were not able to stimulate NSC proliferation (Figure 5(d)). No significant difference between CM-hDPSCs and prCM-hDPSCs could be observed in either assay.

### 3.7. CM-hDPSCs Promotes Neurite Outgrowth of pCNs

**Figure 6:**
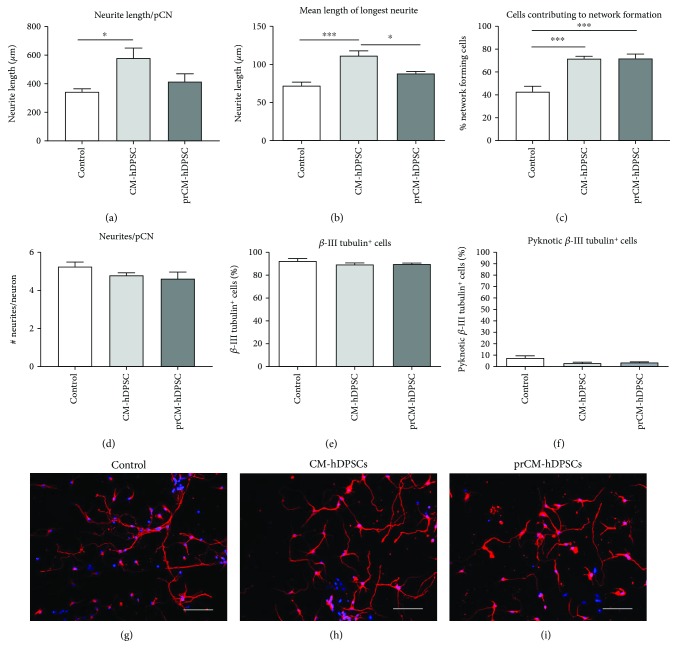
The effect of CM-hDPSCs and prCM-hDPSCs on neurite outgrowth of pCNs. Compared to controls, CM-hDPSCs but not prCM-hDPSCs (*n* = 5) was able to stimulate total neurite outgrowth per neuron (a) and the mean length of the longest neurite (b). The fraction of interconnecting cells was determined as a measurement for cells contributing to network formation (c). These data showed that CM-hDPSCs and prCM-hDPSCs were both able to stimulate neuronal network formation. The effect of CM-hDPSCs and prCM-hDPSCs is not due to an increase in the number of pCN neurites as the number of neurites per pCN is not affected (d). Exposure to CM-hDPSCs or prCM-hDPSCs did not have an influence on the neuronal content of the cell cultures (e) or neuronal death (f) 72 h after exposure as measured by the fraction of *β*-III tubulin-positive cells and pyknotic *β*-III tubulin-positive cells, respectively. Representative micrographs of *β*-III tubulin-stained pCNs after exposure to control medium (g), CM-hDPSCs (h), and prCM-hDPSCs (i). ^∗^*p* value ≤ 0.05; ^∗∗∗^*p* value ≤ 0.001. Data are expressed as mean ± SEM. Scale bars: g–i: 50 *μ*m.

The effect of primed and nonprimed hDPSCs on pCN neurite outgrowth was evaluated by measuring the total neurite length per pCN (Figure 6(a)) and the mean length of the longest neurite (Figure 6(b)). CM-hDPSCs, but not prCM-hDPSCs, was able to stimulate neurite outgrowth in pCNs after 72 h (Figures 6(a) and 6(b)), increasing total neurite outgrowth per neuron from 344.4 *μ*m ± 46.75 *μ*m in controls to 580.5 *μ*m ± 155.6 *μ*m (*p* value: 0.0267) after CM-hDPSCs-exposure. Additional analysis demonstrated that the mean length of the longest neurite increased from 72.15 *μ*m ± 10.35 in controls to 111.7 *μ*m ± 13.78 after CM-hDPSC-exposure (*p* value: 0.0002). The fraction of pCNs that makes contact with one or more neighbouring pCNs was determined as a measure for network formation (Figure 6(c)). After exposure to either CM-hDPSCs or prCM-hDPSCs, pCNs significantly increased the percentage of cells contributing to a network from 42.76%±4.826 in controls to 71.79%±1.994 (*p* value: 0.004) and 72%±3.797 (*p* value: 0.004), respectively. Interestingly, these effects of CM-hDPSCs and prCM-hDPSCs are not due to an increase in the number of neurites per pCN, as these numbers are not significantly altered after exposure of pCNs to both stimulants (Figure 6(d)). Moreover, the fraction of *β*-III tubulin-positive cells (control: 92.56%±1.99) was not significantly altered by adding CM-hDPSCs or prCM-hDPSCs to these cultures, maintaining the neuronal content of the culture (Figure 6(e)). Additionally, to evaluate neuronal survival over the 72 h period, the amount of pyknotic *β*-III tubulin-positive cells was also examined. CM-hDPSCs or prCM-hDPSCs did not have an effect on pCN cell death (Figure 6(f)). Representative micrographs of the control, CM-hDPSC-stimulated, and prCM-hDPSC-exposed pCNs are presented in Figures 6(g)–6(i), respectively.

## 4. Discussion

Plausible mechanisms via which (mesenchymal) stem cells mediate neuronal repair in response to CNS damage include paracrine-induced chemoattraction and proliferation of endogenous NSCs in addition to stimulating the formation of new neuronal connections. This study evaluated paracrine-mediated neuroregenerative mechanisms of hDPSCs on *in vitro* expanded and characterized NSCs and pCNs. In addition, this study was aimed at invigorating the neuroregenerative potential of the hDPSC secretome by preconditioning or priming the cells with L-PRF. This clinically applicable autologous biomaterial was hypothesized to enhance the growth factor secretion pattern of hDPSCs.

In the first phase, the presence of inflammatory mediators of the L-PRF subfractions (CM and EX L-PRF) was determined, and the effect of L-PRF on hDPSCs was evaluated. Both CM and EX L-PRF contained inflammatory mediators such as TNF-*α*, IFN-*γ*, IL-1*α*, IL-1*β*, IGF-1, and IL-10 which were previously shown to influence MSC characteristics [24–28, 30, 31]. In addition, IL-6 and MCP-1 were also found in the L-PRF subfractions and the latter was shown to enhance stem cell-mediated angiogenesis [41]. A detailed analysis of the differentially expressed factors in CM and EX L-PRF can be found in Ratajczak et al. [35] but was out of the scope of this study.

CM and EX L-PRF had an inverse effect on hDPSC metabolism. High concentrations of CM L-PRF significantly decreased the metabolism of hDPSCs, which was increased by high concentrations of EX L-PRF. Both subfractions of the L-PRF secretome stimulated hDPSC proliferation, which can be explained by the platelet-derived components that are present in CM and EX L-PRF as platelet lysates are often used as serum replacement in cell culture [42]. Moreover, it is possible that the concentration of platelet components is higher in EX L-PRF than in CM L-PRF which can explain the larger effect of EX L-PRF on hDPSC proliferation than CM L-PRF. Furthermore, the presented data suggest that the observed decrease in metabolic activity is an effect of CM and EX L-PRF on hDPSC metabolism, as no effect of the L-PRF subfractions on cell death was observed in the PI assay. Moreover, based on the semiquantitative analysis of the inflammatory mediators in CM and EX L-PRF, no significant difference in the level of mediators between both subfractions could be detected. This suggests that the observed differences in metabolic activity between CM and EX L-PRF-treated hDPSCs are not due to the level of the (analysed) inflammatory mediators. In addition, these contradicting results using the MTT- and PI assay emphasise that both tests are inherently different and have different readout parameters although the MTT test is often used as an assay to measure cell proliferation. A reason for the discrepancy in metabolic activity is unknown. Candidate factors might include those components that alter mitochondrial function and subsequently the MTT assay such as leukocyte-derived myeloperoxidases [43], TNF-*α* [23], and IL-1*β* [22] which lead to reactive oxygen species production [44].

Next, hDPSCs were primed with different concentrations of CM and EX L-PRF and BDNF secretion was taken as a measure for increased neurotrophin production. Priming hDPSCs for 24 h or 48 h with 10% CM or EX L-PRF significantly enhanced BDNF production by hDPSCs. However, due to the negative effect of CM L-PRF on hDPSC metabolism, 10% EX L-PRF was subsequently used to prime hDPSCs.

The proliferative and chemoattractant properties of CM-hDPSCs and prCM-hDPSCs on NSCs were evaluated next. The NSCs applied in this study showed the immunophenotype for the markers described in Reekmans et al. [37]. In accordance with previous data on the effect of CM-hDPSCs on SH-SY5Y cells [14], CM-hDPSCs did not influence NSC proliferation or metabolic activity and priming hDPSCs with L-PRF did not alter this response. CM-hDPSCs and prCM-hDPSCs were used to evaluate their chemoattractant properties on NSCs in a transwell assay as recruitment of endogenous NSC to the site of neuronal injury is one of the goals in neuroregenerative medicine. CM-hDPSCs significantly promoted NSC migration but again, this ability was not improved by L-PRF priming. Factors secreted by hDPSCs that can be responsible for this chemoattractive effect on NSCs include MCP-1 [45, 46], IL-8 [45, 47], and SDF-1 [48].

Finally, the neuritogenic potential of the secretome of primed and nonprimed hDPSCs on pCNs was evaluated as these are key aims for therapies targeting CNS regeneration. CM-hDPSCs but not prCM-hDPSCs was able to promote neuritogenesis in pCNs compared to controls in standard pCN medium. Moreover, both conditioned media did not significantly influence the purity of the neuronal culture or the number of pyknotic cells, excluding cytotoxic effects of the conditioned media. The factors responsible in the CM-hDPSCs for enhanced neurite outgrowth include the factors working via tyrosine receptor kinases (Trk). These include but are not limited to nerve growth factor, NT-3, BDNF, glial-derived growth factor, and VEGF [7, 32, 49].

Despite the efforts that were made to enhance the secretome of hDPSCs by L-PRF priming, no additional effect on neuroregenerative mechanisms was observed by priming hDPSCs with L-PRF. It can therefore be concluded that L-PRF priming does not alter the hDPSC secretome to a more favourable composition for neuroregeneration. Nonetheless, the priming effect on distinct fields of regenerative medicine remains to be elucidated. L-PRF components such as MCP-1, IL-8, and TNF-*α* were previously shown to enhance MSC-mediated angiogenesis [27, 41, 50] whereas IL-10, IFN-*γ*, IL-*α*, and IL-1*β* were shown to stimulate the immunomodulatory properties of MSCs [28, 30, 31]. Future hDPSC priming studies with the cocktail of growth factors present in the L-PRF subfractions should therefore focus on other regenerative mechanisms associated with wound healing. Moreover, it should be noted that in this study, a fairly young population of hDPSC donors was used (14-26 years of age) which were able to produce conditioned medium with neuroregenerative effects. Considering that autologous stem cell therapies should also be applicable for the elderly population, this raises concern on the potency of hDPSCs derived from older donors to produce comparable conditioned medium. While this was out of scope of this study, contradicting studies on the matter of age-related differences in hDPSCs are available. On the one hand, Feng et al. and Ma et al. demonstrated that age decreases the proliferation rate, migration, and osteogenic differentiation potential of DPSCs [51, 52]. On the other hand, several studies report that age does not influence the gene expression profile of DPSCs and that the stem cell properties and regenerative potential of DPSCs are independent of donor age [53–55]. Despite these reports that suggest that age does not influence the regenerative potential of hDPSCs, no information on age-related differences in the composition of CM-hDPSC is available.

A final remark that can be made is that we demonstrated that L-PRF subfractions, and more specifically EX L-PRF, stimulate hDPSC proliferation when used as a medium supplement comparable to standard culture conditions using FCS. This is of particular interest as ideally, stem cells for clinical grade applications should be cultured in xeno-free conditions that significantly stimulate cell growth to obtain sufficient cell numbers in a short time period due to the genetic instability that arises in long-term cell culturing [56, 57]. Additionally, a recent report by Haque and Abu Kasim demonstrated that the use of FCS can influence the composition of the conditioned medium derived from stem cells from human extracted deciduous teeth compared to the secretome of these cells cultured in human serum [58]. Therefore, due to the ease and speed of blood-derived EX L-PRF isolation, it offers a potential autologous serum replacement strategy to supplement the culture medium that is needed for the *in vitro* expansion period that is often required before sufficient autologous stem cell numbers can be obtained for regenerative purposes. These findings are in line with Greiner et al., who demonstrated the successful use of human blood plasma as an additive for the culture of human neural crest-derived inferior turbinate stem cells which in addition did not influence the stem cell characteristics and differentiation potential of these cells [57]. Therefore, future studies that are aimed at using (EX) L-PRF as a serum replacement strategy for standard stem cell culture should evaluate the prolonged effect of L-PRF subfractions on the genetic stability, secretome composition, the stem cell properties, and differentiation potential of hDPSCs. However, this was beyond the scope of the present study.

In conclusion, the present study demonstrated the paracrine-mediated neuroregenerative potential of hDPSCs on NSC proliferation and migration and on stimulating pCN neurite outgrowth. Moreover, the effect of preconditioning hDPSCs with L-PRF which was hypothesized to improve the neuroregenerative potential of the hDPSC secretome was investigated. This approach has to our knowledge not been used before. The results in this study demonstrated that the CM of hDPSCs was capable to enhance neuritogenesis in pCNs. Moreover, this CM had a chemoattractant effect on NSCs but did not stimulate NSC proliferation. Although priming hDPSCs had no additional effect on the paracrine-mediated mechanisms of neuroregeneration described in this study, the potential of L-PRF-primed hDPSCs on distinct regenerative mechanisms remains to be clarified.

## Figures and Tables

**Table 1:** Primary and secondary antibodies for immunocytochemical analysis.

Marker	Species	Clone, Cat no.	Dilution	Label	Company
*Primary antibodies*					
A2B5	MM	MAB312R	1/200		Millipore, Billerica, MA, USA
BLBP	RP	ABN14	1/250		Millipore
*β*-III tubulin	RP	ab18207	1/50		Abcam, Cambridge, UK
*β*-III tubulin	MM	2G10	1/2000		Sigma-Aldrich
CD45	RP	Ab63390	1/100		Abcam
GFAP	MM	Clone G-A-5	1/400		Sigma-Aldrich
NCAM	RP	Ab5032	1/100		Millipore
NeuN	MM	A60, MAB377	1/100		Millipore
Sca-1	GP	AF1226	1/50		R&D, Minneapolis, MN, USA
Sox2	RP	Ab97959	1/1000		Abcam
*Secondary antibodies*					
DAM	DP IgG	A31570	1/500	Alexa Fluor 555	Invitrogen, Carlsbad, CA, USA
DAR	DP IgG	A21206	1/500	Alexa Fluor 488	Invitrogen
DAG	DP IgG	A11055	1/500	Alexa Fluor 488	Invitrogen

BLBP: brain lipid-binding protein; GFAP: glial fibrillary acidic protein; NCAM: neural cell adhesion molecule; NeuN: neuronal nuclei; Sca-1: stem cell antigen-1; MM: mouse monoclonal antibody; RP: rabbit polyclonal antibody; GP: goat polyclonal; DP: donkey polyclonal; DAM: donkey anti-mouse; DAR: donkey anti-rabbit; DAG: donkey anti-goat.

## Data Availability

The datasets analysed during the current study are available from the corresponding author on reasonable request.
